# Supplementary material for: Real-World evidence revelations: The potential of patient support programmes to provide data on medication usage
Source: PLoS One. 2024 Feb 8;19(2):e0295226. doi: 10.1371/journal.pone.0295226 (PMC10852303; doi:10.1371/journal.pone.0295226)
Supplement: S1 Annex — (DOCX) [file pone.0295226.s002.docx]

**Annex**

**Sacubitril/valsartan reported cases of "tablet splitting" / "once-daily dose" case numbers per country**

| Counties | Case numbers of reported cases on "tablet splitting" / "once-daily dose instead twice" case numbers |
| --- | --- |
| Argentina | 55 |
| Australia | 15 |
| Bangladesh | 2 |
| Belgium | 2 |
| Brazil | 64 |
| Canada | 12 |
| Chile | 2 |
| China | 16 |
| Colombia | 392 |
| Costa Rica | 84 |
| Cyprus | 1 |
| Dominican Republic | 34 |
| Ecuador | 22 |
| Egypt | 151 |
| El Salvador | 1 |
| Germany | 5 |
| Guatemala | 171 |
| Honduras | 118 |
| Hungary | 1 |
| Iceland | 1 |
| India | 4776 |
| Korea, Republic Of | 72 |
| Lebanon | 85 |
| Lithuania | 2 |
| Malaysia | 13 |
| Mexico | 1809 |
| Morocco | 1 |
| Pakistan | 60 |
| Panama | 22 |
| Peru | 3 |
| Philippines | 156 |
| Portugal | 1 |
| Puerto Rico | 1 |
| Russian Federation | 11 |
| Switzerland | 6 |
| Thailand | 156 |
| United Arab Emirates | 5 |
| United Kingdom | 33 |
| United States | 1304 |
| Grand Total | **9665** |
