# Supplementary material for: Real-World evidence revelations: The potential of patient support programmes to provide data on medication usage
Source: PLoS One. 2024 Feb 8;19(2):e0295226. doi: 10.1371/journal.pone.0295226 (PMC10852303; doi:10.1371/journal.pone.0295226)
Supplement: Attachment — Submitted filename: Project Proposal_2019 template_final_29April2020.doc.pdf [file pone.0295226.s003.pdf]

DocuSign Envelope ID: D8283142-F48D-49D3-952D-2CCE14EEDAA3

Appendix 1

University of Hertfordshire

School of Life and Medical Sciences

MSc Pharmacovigilance Project Proposal

1. General information

	Student	Workplace supervisor
**Name and SRN**	Eszter Palffy	David Lewis
**Job title**	Country Patient Safety Head	EU QPPV
**Department and address**	Global Drug Development<br><br>Novartis Ireland Limited<br>The Vista Building<br>Elm Park Business Park<br>Merrion Road, Dublin 4<br>D04 A9N6, Ireland	Global Drug Development<br><br>Novartis Pharma GmbH<br>Oeflinger Strasse 44<br>D-79664 Wehr
**Telephone**	Private: 00 353 87 168 3090<br>Work: 00 353 87 762 1116	T +49 7762 82 3010<br>M +41 79 582 3859
**e-mail**	Eszter.palffy@novartis.com/<br>palffyezu@yahoo.com	david-1.lewis@novartis.com

2. Project title

The evolution of the Governance of Patient Support Programmes at Novartis – and how do PSPs contribute to signal detection?

3. Project rationale *(800 words; this should also include the expected outcome.)*

The framework EU Pharmacovigilance Legislation was issued in June 2012 (Arlett P, 2012). Prior to the GVP modules, Volume 9A did not provide detailed governance for all areas of PV systems. One of areas of poor coverage was the solicited programmes especially Patient Support Programmes. The new legislation, and the

DocuSign Envelope ID: D8283142-F48D-49D3-952D-2CCE14EEDAA3

supporting guidance in Good Vigilance Practice Module VI (EMA, 2017), better defines and governs these programmes. As defined, a PSP is an organised system where the marketing authorisation holder (MAH) receives and collects information relating to the use of its medicinal products. The programmes can be post-authorisation patient support and disease management programmes, surveys of patients and healthcare providers, information gathering on patient compliance, or compensation/ re-imbursement schemes.

Under GVP module VI, these programmes are considered solicited reports and the MAH is responsible for collecting and evaluating full and comprehensive case information in order to asses and report individual cases related to the studied (or supplied) medicinal product. The MAH should request causality assessment from the primary reporter of each case report, and where possible and consented an appropriate level of due diligence should be exercised by the MAH.

Since 2012, the governance of PSPs has evolved. However, the underlying question, whether such efforts from the MAH leads to any newly identified signals on the medicinal product, is still not clear. Limited evidence is available in the public domain (e.g. Portnoff JM et al. 2017; Jokinen JD et al. 2019), but more research is required to determine if there has been an impact of the 2012 PV legislation on patient safety. Indeed there is a bigger question concerning whether there have been any tangible public health benefits from the significant investment of time and resources applied by MAHs to govern and manage PSPs.

Jokinen et al. is the first multi-company collaborative effort using statistical methods to analyse the different sources of safety data i.e. solicited and spontaneous reports combined for signal detection. The conclusion was that the increased volumes of safety data from PSPs do not necessarily correlate with improved safety signal detection. The paper also suggests an over 400% increase in AE reporting in a decade due to PSPs, market research and social media programmes. None of these programmes are designed to collect AEs however; PSPs do generate a great number of AEs due to their nature. PSPs are structured to provide support in different forms of services, solutions or initiatives to assist patients in managing their disease or medication. This finding further strengthens the hypothesis and research question whether this reporting from PSPs leads to any validated signals that further strengthens knowledge of the safety profile of the medication.

Portnoff et al. researched the governance and oversight of PSPs at the MAH level. PSPs fall under the scope of “Organized data collection systems” as classified by

DocuSign Envelope ID: D8283142-F48D-49D3-952D-2CCE14EEDAA3

GVP module VI and obliges the MAH to collect AEs deriving from such programmes. However, there are no further guidance available and each MAH must determine how to ensure AE collection and reporting. The paper revealed that most companies reported “regular oversight” of PSPs and other organised data collection systems with few companies admitting lesser oversight. The question remains what a regular oversight is as it may be interpreted differently.

Neither of the papers however, analysed if such effort from the MAH on collecting and reporting AEs from PSPs in fact add any value to the actual signal detection process considering the fact that such programmes are not designed for AE collection. Currently at Novartis, there is a robust system in place with several global and local processes to ensure the appropriate management and oversight of PSPs.

Expected outcome

The expected outcome is that PSPs will not generate any signals as they are not intended to collect AEs and there are no written protocol or hypothesis to examine. PSPs however, can be beneficial for reviewing the acceptance, adherence and tolerability and many more other aspects of participating patients and healthcare professionals.

Regulators should assess the time and effort spent by the MAH on collection and reporting of adverse events from PSPs in comparison to the benefit and information provided from these reports to ensure useful investment for case handling for public health reason. Does it really add value to identifying new signals or could resources be deployed in other much more useful methods? Regulators might need to reassess the process of individual case submission from PSPs to a more beneficial periodic report submission with focus on patient and health care professional experience.

4. Aims and Objectives *(please list)*

The aim of the research is to evaluate the evolution of PSPs within Novartis and further assess if the 2012 EU PV legislation supports the pharmacovigilance and signal detection of the medicinal products involved in PSPs. In general, PSPs do not need scientific hypothesis or governed by a protocol hence these programmes are not safety oriented. However, the nature of these programmes generate reportable AEs. The project will also review the benefit of collection and reporting of adverse events from PSPs in comparison to the time and effort spent by the MAH to fulfil the current regulations.

DocuSign Envelope ID: D8283142-F48D-49D3-952D-2CCE14EEDAA3

This review and assessment will be achieved by focusing on the wide range of PSPs in association with two Novartis innovative medicines, Cosentyx (secukinumab) and Entresto (Sacubitril/valsartan); and the development of their safety profile.

The justification for the selection of Cosentyx and Entresto is that the Novartis publication policy supports the release of additional information in the public domain after a defined period.

Cosentyx was first registered in Japan on 24th Dec 2014 (International Birth Date or IBD). It was approved by the European Medicines Agency (EMA) on 14th January 2015, followed by the US Food and Drug Administration (FDA) on 21st January 2015. To date Novartis has obtained approvals in 92 countries worldwide Cosentyx is registered to treat moderate to severe plaque psoriasis, psoriatic arthritis, ankylosing spondylitis and pustular psoriasis (in Japan only). The formulation of Cosentyx is a pen or prefilled syringe containing 150 mg of secukinumab for subcutaneous administration.

Entresto was first approved and registered in the United States of America (USA) on 7th Jul 2015 (IBD), followed by the EMA approval on 25th Sep 2015. Novartis is currently the MAH in 111 countries worldwide. Entresto is registered for the treatment of symptomatic chronic heart failure with reduced ejection fraction (HFrEF) in adult patients.

Over the past five years, in the PSP database (POPsys) there are over a hundred PSPs on Cosentyx and over 70 on Entresto. This is likely to provide sufficient information for analysis. This time period is sufficient for Novartis to allow meaningful use of the data.

The two chosen products have very different profiles, e.g. indication, formulation, target group, therapy areas etc. However, both have been on the market roughly the same time. It would be interesting to see if PSPs have contributed to signals.

Additionally, the project will look deeper into the PSPs from the patient perspective. Since patients can report adverse events via the support programmes there is a higher possibility to receive and collect patient tolerability information. Tolerability is a preference whereby the patient is able to tolerate a certain level of AEs to continue treatment, e.g. non-serious patient reported tolerability of the product in comparison to unexpected adverse events. The project will further look into patient adherence to and acceptance of the product.

DocuSign Envelope ID: D8283142-F48D-49D3-952D-2CCE14EEDAA3

The intended objectives includes the following:

- To review the available literature on the evolution of PSPs: the major changes and observed improvements post 2012.
- To review the Patient Support Programmes created at Novartis from 2012 globally focusing on the two innovative products, Cosentyx (secukinumab) and Entresto (sacubitril/valsartan).
- To review the patterns provided by the cohort of cases entered into the safety database (ARGUS) from 2012 on PSPs involving Cosentyx and Entresto.
- To assess if there have been any signals raised and validated from these programmes (PSPs only).
- To review if PSPs contribute to any new signals or could be used for other purposes.

5. Main hypothesis/research question addressed in the project *(1-4 sentences)*

The current legislation requires all AEs to be collected and reported by the MAH from all PSPs and this leads to over reporting. The question arises whether this heavy reporting from PSPs leads to any validated signals that further strengthens knowledge of the safety profile of the medications. On the other hand, could this information from PSPs provide a good data source for other purposes e.g. patient tolerability, acceptance, and adherence to the product?

6. Plan of the investigation and timing *(max. 500 words)*

Plan of investigation: Nine months in total
Start of the project: Feb 2020
Finish of the project: approx. Dec 2020
Submission of project: Dec 2020

Project timeline:

- Research of background, introduction and plan of methods – 2 months
- Gather data and evaluation of results – 2 months
- Discussions and conclusions of data and further research if needed – 3-4 months

DocuSign Envelope ID: D8283142-F48D-49D3-952D-2CCE14EEDAA3

- Write-up of research, references, formatting + critique of research – 1-2 months

7. Intended statistical analysis *(max. 250 words including the rationale)*

The statistical part of the research will be descriptive statistics. It is planned to review how the safety profile changed i.e. how many newly identified adverse drug reactions, if any, have been added to the label since the product was launched.
A database review of POPsys (PSP database) and ARGUS (safety database) will be carried out. The adverse event line listing on all Cosentyx and Entresto PSPs will be compared to the label changes from the launch of Cosentyx and Entresto.
This comparison will provide evidence as to whether any serious or non-serious AEs reported through the Cosentyx/Entresto PSPs worldwide contributed to any validated signals for the product.

8. References *(list 4 to 6 key references used to develop the project)*

Arlett P (2012). The new European Union pharmacovigilance legislation.
Accessed on 20-Nov-2019 from https://www.youtube.com/watch?v=aCmrV3fS-4Y.

European Medicine Agency (2017). Guideline on good pharmacovigilance practices (GVP) Module VI – Collection, management and submission of reports of suspected adverse reactions to medicinal products (Revision 2). Accessed on 20-Nov-2019 from
https://www.ema.europa.eu/en/documents/regulatory-procedural-guideline/guideline-good-pharmacovigilance-practices-gvp-module-vi-collection-management-submission-reports_en.pdf.

Portnoff JM and Lewis DJ (2017). The enigma of pharmacovigilance of patient support programmes: a survey of marketing authorisation holders in Europe; Therapeutic Innovation & Regulatory Science; 51(4): 486-93. doi: 10.1177/2168479017696264.

Jokinen JD, Walley RJ, Colopy MW, Hilzinger TS, Verdru P (2019). Pooling different safety data sources: impact of combining solicited and Spontaneous Reports on Signal Detection in Pharmacovigilance. Drug Safety; 42(10):1191-1198. doi: 10.1007/s40264-019-00843-0.

Jokinen J, Bertin D, Donzanti B, Hormbrey J, Simmons V, Li H, Dharmani C, Kracht K, Hilzinger TS, Verdru P (2019). Industry Assessment of the contribution of Patient Support Programs, Market Research Programs, and Social Media to Patient Safety; Therapeutic Innovation & Regulatory Science; 53(6) 736-745. DOI: 10.1177/2168479019877384

9. Ethical considerations for human subjects

All PSP participants have given consent to take part in the programme as well as consented to adverse event reporting.
The data used for the research will be presented as cohort only with no identification of individual patients; therefore, there will be no data privacy breach.

Word limit: 2000

10. Signature

DocuSigned by:
Eszter Palffy
19C476E825054F2...
30-Apr-20 | 3:14:30 PM BST

DocuSigned by:
Dr David J Lewis
CF07B9282B124B1...
30-Apr-20 | 4:33:14 PM GMT

DocuSigned by:
John Talbot
7A2488C5DB30421...
30-Apr-20 | 6:58:57 PM BST

Eszter Palffy	David J Lewis	John Talbot
**Student**	**Workplace**	**University**
