# Supplementary material for: Real-World evidence revelations: The potential of patient support programmes to provide data on medication usage
Source: PLoS One. 2024 Feb 8;19(2):e0295226. doi: 10.1371/journal.pone.0295226 (PMC10852303; doi:10.1371/journal.pone.0295226)
Supplement: Attachment — Submitted filename: Letter from Chair of Ethics.pdf [file pone.0295226.s004.pdf]

University of Hertfordshire
Hatfield
AL10 9AB
UK

tel +44 (0)1707 284000
herts.ac.uk

07/06/2023

To Whom It May Concern,

Your reference: Manuscript Number - PONE-D-22-27481R1

I write to confirm that the above research study did not require ethical approval as it did not involve the use of Human Participants.

The study analysed secondary data therefore did not fall within the scope of our policy (RE01-Studies-Involving-Human-Participants.pdf (herts.ac.uk)).

Yours Sincerely

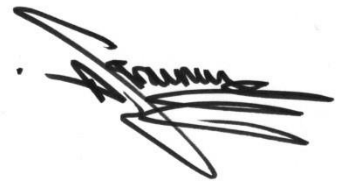

Dr Simon Trainis
Chair Ethics Committee with delegated authority for Health, Science, Engineering and Technology

S.A.Trainis@herts.ac.uk

A Charity Exempt from Registration under the
Second Schedule of the Charities Act 1993
