# Supplementary material for: Real-World evidence revelations: The potential of patient support programmes to provide data on medication usage
Source: PLoS One. 2024 Feb 8;19(2):e0295226. doi: 10.1371/journal.pone.0295226 (PMC10852303; doi:10.1371/journal.pone.0295226)
Supplement: Attachment — Submitted filename: Response to Reviewer_ver.1.0_02Nov2023.docx [file pone.0295226.s005.docx]

**We believe that Reviewers #1, #4 and #5 were satisfied with the content of the manuscript and we have addressed the points made by Reviewer #3.**

- Title of study is not informative. You should indicate the study design and location in title.

A. We have proposed a new title – see revision.

- In abstract, you should clarify your main goal.

A. Main goal clarified in paragraph 2 of the abstract.

- Introduction is too complex. Please concise it.

A. Four reviewers have supported the current text; one has raised comments. As this is a complex topic, we prefer to preserve the majority of our introduction. Nevertheless, we have reduced the text where possible.

- In introduction, you should specify current gaps and clarify your main goals.

A. Gaps are highlighted within the new text on the inspection history. The main goals of our research have been clarified in the abstract.

- You should explain your methods in more details so that any researcher be able to repeat it.

A. We have described in the Materials section the products, the programmes, the existing reference documentation, and the evidence gathered, as well as the processes and the POPSys database. Within the Methods section, we have described our research. In our view this research may be repeated by others who have access to data from PSPs.

- Table 4 is not necessary. You can insert such information in the text.

A. We wish to request that Table 4 is placed as an annex to the paper. We have added text to allow us to remove this table into an annex, so that key information is visible to readers, thus we feel that we have provided an appropriate response to this comment.

- In the first paragraph of discussion, you should report your main findings.

A. Paragraph moved to clarify main (unexpected) finding at the start of the Discussion.

- Your conclusion is too long.

A. We were asked to add three paragraphs to the Conclusion by three other reviewers, hence we are reluctant to remove text at this stage, as it would displease the majority of the reviewers. The topic requires care, such that we do not dismiss real world evidence provided directly by patients. Nevertheless, we have reduced the text where possible.
